# The Rise of ‘Group Solution’ in Insolvency Law and Bank Resolution

**DOI:** 10.1007/s40804-021-00220-4

**Published:** 2021-07-29

**Authors:** Ilya Kokorin

**Affiliations:** 1 Department of Financial Law, Leiden University, Leiden, The Netherlands; 2 Member of Class IX, III NextGen Leadership Program, Stanardsville, VA USA; 3 Committee member, INSOL International’s Early Researcher Academics (ERA), London, UK; 4 Member of the Young Researchers Group, European Banking Institute (EBI), Frankfurt am Main, Germany

**Keywords:** Group solution, Insolvency law, Bank resolution, Enterprise groups, No creditor worse off principle, UNCITRAL Model Law, European Insolvency Regulation, BRRD

## Abstract

This article traces the emergence of the concept of ‘group solution’ and its manifestations in insolvency law and bank resolution as an alternative to the rigid entity-by-entity approach. The rise of this concept can be linked to the recognition of the specificity of problems related to the insolvency of multinational enterprise groups, arising from group operational and financial interconnectedness. This has not happened at once, but has resulted from the evolution of views and ideas, evident in hard and soft law instruments of the 2000s and the 2010s. In light of this important development the article explores the concept of a group solution, its rationale, scope of application and limitations. It concludes that despite the gradual acceptance of the group phenomenon, a group solution has not been formed as a coherent and well-defined legal principle. Instead, it represents a variety of approaches, tools and practices, which pursue different policy objectives underpinned by different societal values. Among them are asset value maximization, business rescue, the protection of financial stability and the preservation of banks’ critical functions. With all its flexibility, a group solution has one pervasive limitation—it cannot trump the interests of individual group members and their creditors. At the same time, in order to realize the full potential of a group solution, it is necessary to embrace the group-sensitive and forward-looking interpretation of creditors’ interest, facilitating commercially sensible and practical group solutions.

## Introduction

The outbreak of COVID-19 and the subsequent drastic governmental measures to curb the pandemic have affected many businesses, small and large. It may be too early to fully grasp the consequences of COVID-19 and determine its long-term effects (if any) on the foundations of insolvency law.^1^ The pandemic has severely affected international trade, bringing the world economy to a near standstill and intensifying ‘territorialist’ sentiments.^2^ This particularly hits multinational enterprise groups, dependent on global supply chains and uninterrupted liquidity flows (e.g. airlines, automotive manufacturing). In the years to come we may expect an increase in the number of international group insolvencies, spanning across various jurisdictions and affecting networks of many smaller businesses. Does insolvency law offer a solution?

Insolvency law has traditionally been used as an instrument to resolve creditors’ claims in the most efficient way. This is done through a collective procedure, which replaces individual creditor action. A court supervises the use and disposition of assets and holds them together to preserve and maximize their value.^3^ For a long time (and perhaps still), insolvency law has largely remained microprudential or single entity-focused, resulting in the entity-by-entity treatment of enterprise group members in financial distress. It is therefore primarily centred around post-crisis liquidation of the debtor’s assets and the allocation of sale proceeds among creditors of an individual legal entity.

However, modern insolvency law increasingly acknowledges the specificity of enterprise group insolvencies, premised on the existence of close operational and financial links and interdependencies between group members. It recognizes that the entity-by-entity approach often leads to the loss of group synergies, group disintegration and suboptimal outcomes for creditors, debtors and other stakeholders (e.g. employees).^4^ As a result, since some two decades, tailored rules and recommendations addressing enterprise group insolvency have been proposed or adopted at the national (Germany), regional (European Insolvency Regulation, recast)^5^ and international (UNCITRAL Model Law on Enterprise Group Insolvency)^6^ levels. Recognition of corporate groups has gradually taken place both in insolvency law and in the area handling the failure of credit institutions and banking groups—bank resolution (Bank Recovery and Resolution Directive).^7^

The accumulation of knowledge and the practice of dealing with multinational groups of companies in restructuring, insolvency and resolution justify an examination of these new legal instruments and developments. Specifically, this article explores whether a new phenomenon or even a legal principle of a ‘group solution’ (as opposed to an ‘entity solution’) has taken shape and has replaced or pushed aside the inflexible entity-by-entity approach. This examination is carried out by this article, which analyses five main questions: (i) what is a group solution?; (ii) which goal does a group solution seek to achieve?; (iii) how does it try to achieve it?; (iv) what are the limitations or boundaries of a group solution?; and (v) what are the strengths and weaknesses of modern approaches to a group solution?

The article proceeds as follows. Section 2 describes the evolution of approaches to corporate groups in insolvency law and highlights the persistent discrepancy between group economic reality and the legal responses to financial distress. Section 3 introduces the concept of a ‘group solution’ and explains its origin, rationale and major limitations in insolvency law. Section 4 illustrates how a group solution manifests itself in the context of the resolution of financial institutions and cross-border banking groups. It discusses the goals of bank resolution (4.1), group recovery and resolution plans (4.2), intragroup financial support agreements (4.3) and different resolution strategies (4.4). It also highlights the limits of group resolvability (4.5). Section 5 analyses the norms related to substantive consolidation, pointing out that such consolidation should not be considered a group solution *stricto sensu*. Section 6 provides a possible economic explanation for a group solution and calls for a broad interpretation of creditors’ interests. Section 7 questions whether a group solution forms a stand-alone legal principle and argues that it has not yet developed into a well-defined legal principle. Instead, it plays an important supporting and facilitating role. Section 8 concludes.

## Recognition of a Corporate Group in Insolvency

### The Long-Standing Entity-by-Entity Approach

Until recently the problem of the insolvency of corporate groups has not been widely recognized or addressed in hard and soft law. Thus, both the UNCITRAL Model Law on Cross-Border Insolvency (Model Law 1997),^8^ the Directive on the reorganisation and winding up of credit institutions (CIWUD)^9^ and the original European Insolvency Regulation (EIR 2000)^10^ lack provisions addressing enterprise group insolvency. The explanation given by the UNCITRAL Working Group V, responsible for drafting the text of the Model Law 1997, is quite revealing. It stated that ‘[w]hen the text of what became the UNCITRAL Model Law on Cross-Border Insolvency (Model Law) was debated, groups were regarded as “a stage too far”.’^11^ This explanation can be easily extended to the European Union (EU) context. There are a number of reasons for the slow acceptance of corporate groups in insolvency.

First, one would expect that within European company and insolvency law special rules addressing groups of companies would have been developed, but the tension between the prevailing entity-by-entity approach and the economic reality of enterprise groups is not easy to tackle.^12^ The entity-by-entity treatment of group entities in insolvency is firmly grounded in the long-established principle of entity separateness.^13^ Its application in insolvency means that: (i) assets in an enterprise group are separated along the entity lines (one entity—one insolvency estate), (ii) creditors have legal rights (recourse) only with respect to particular entities and their assets, and (iii) insolvency practitioners appointed in individual companies act in the interests of the respective companies and their creditors. Wessels and Madaus call this a ‘five one’s’ principle as it establishes ‘one insolvent debtor, one estate, one insolvency proceeding, one court and one insolvency office holder’.^14^ The fact that before insolvency the group operated as one integrated enterprise does not easily fit this atomistic vision.

Second, the very concept of a ‘group of companies’ has not been comprehensibly developed or harmonized across company laws. While some jurisdictions apply general corporate and/or civil law to enterprise groups (e.g. the UK), others have adopted special rules (e.g. Germany, Portugal, Slovenia).^15^ Nevertheless, there is some convergence of approaches when it comes to agency problems arising in corporate groups, including conflicts between majority and minority shareholders, and shareholders and creditors. This convergence manifests itself in the rules concerning related-party transactions. In the vast majority of the EU Member States, but also outside the EU, company and insolvency laws establish special treatment for transactions involving related parties—shareholders and their affiliates, sometimes referred to as insiders. In company law this results in the safeguards against intragroup self-dealing, including the allocation of certain rights to minority shareholders (e.g. appraisal rights), disclosure and approval requirements.^16^ In insolvency, the interests of insiders usually enjoy lesser, minimal or no protection against transaction avoidance. In practice this is evident in the extension of suspect periods,^17^ the acceptance of certain mental elements (e.g. knowledge or intention to give preference or to defraud)^18^ or a rebuttable presumption of harm caused to creditors.^19^

However, the recognition of corporate groups faces significant obstacles and even resistance whenever the principle of entity separateness is at stake. A good example is the discussion about the recognition of ‘group interest’ in European company law that has taken place since the 1990s, first solely among academics^20^ and then under the EC Consultation on the Future of European Company Law launched in 2012. The latter resulted in the Company Law Action Plan, which included an initiative to recognize the concept of a group interest.^21^ Despite the generally positive attitude of scholars and the business community on the EU-wide move towards the acceptance of ‘group interest’,^22^ this initiative has not led to any legislative proposals, highlighting its complex and controversial character. The idea of a comprehensive legal EU framework covering groups of companies was also met with caution.

### From a Single Entity to a Single Enterprise

The single entity approach relies on the principle of legal separability or entity separateness. Thus, insolvency estates and pools of creditors of legal entities are separated. On the one hand, this limits the risks attached to a failure of a single company, since creditors of one debtor do not automatically become creditors of all other group members. In theory this allows for better risk calculation, since it is easier to evaluate a borrower’s creditworthiness.^23^ On the other hand, the economic interconnectedness of group companies, as well as various intragroup liability arrangements (e.g. intragroup loans, cross-guarantees, cross-default provisions and cross-entity ipso facto clauses) highlight the unsophistication of this approach and could make it less appealing.^24^ To remedy these deficiencies, the enterprise approach has been suggested. This approach treats the group as a single economic unit that operates to further the interests of the group as a whole.^25^ The enterprise approach is visible in a variety of rules and techniques, from less intrusive (e.g. communication and cooperation between insolvent group members) to the ultimate disregard of entity boundaries (i.e. substantive consolidation).^26^

Recent years have witnessed the rise of important initiatives and the emergence of new legal instruments that modernize insolvency law to ensure an effective and efficient administration of cross-border insolvency proceedings in the context of groups of companies. Among such instruments are the BRRD (2014), the EIR Recast (2015), and the UNCITRAL Model Law on Enterprise Group Insolvency (2019). The BRRD, the EIR Recast and the Model Law 2019 recognize the specificity of corporate groups, give a definition of a group of companies^27^ and offer (and sometimes mandate) special mechanisms and tools to address the challenges of enterprise group insolvencies.

A number of national legal regimes also contain regulations on enterprise group insolvency. For example, Germany reformed its insolvency law in 2017 to facilitate the efficient administration of insolvency proceedings through enhanced coordination. This is achieved by means of the concentration of insolvency proceedings in one court, the appointment of the same insolvency practitioner in separate insolvency proceedings and the opening of special group coordination proceedings (*Koordinationsverfahren*).^28^ France, Italy, Belgium and Spain have adapted their general insolvency rules to provide for the joint commencement of proceedings in cases of enterprise groups.^29^ The English schemes of arrangement are frequently used to restructure debts of various group members at the same time, giving effect to a group-wide solution.^30^ This is particularly evident from the sweeping approval by the English courts of so-called third-party releases, which lead to a release (i.e. a total or partial discharge or amendment) of claims against third parties, such as co-obligors, guarantors and collateral providers (typically, group members) in the proceeding concerning the principal debtor.^31^ In the case of *Syncreon Group BV*, the court noted that such releases were necessary ‘in order to give full effect to the schemes’ and that they were ‘a relatively regular feature of the schemes’.^32^

The new generation of legal instruments mentioned above recognises the need to adjust traditional tools of insolvency law to the characteristics and business reality of corporate groups. However, the extent of such recognition and its boundaries are not entirely clear.

## The Rise of ‘Group Solution’ in Insolvency Law

### Group Solution and Asset Value Maximization

The EIR Recast establishes that cooperation in the context of enterprise group insolvency ‘should be aimed at finding a *solution* that would leverage synergies across the group’.^33^ In a like manner, the Model Law 2019 aims to provide effective mechanisms to address cases of the insolvency of groups of companies through facilitation of the development of *group insolvency solutions* for the whole or part of an enterprise group.^34^ A group insolvency solution is defined as:

A proposal or set of proposals developed […] for the reorganization, sale or liquidation of some or all of the assets and operations of one or more enterprise group members, with the goal of protecting, preserving, realizing or enhancing the overall combined value of those enterprise group members.^35^

As explained in the Guide to Enactment of the Model Law 2019, a group insolvency solution is a new term, which is intended to be flexible.^36^ This flexibility is needed to take into account the circumstances of a specific enterprise group, its corporate and financial structure, business model, as well as the degree of integration between different group members.

For integrated groups of companies, a group solution often entails the preservation of going concern value, which requires the prevention of group disintegration upon insolvency. Imagine the following (rather typical) scenario. A complex corporate group consists of a number of entities playing different roles: a company issuing debt instruments and lending the received funds to other group members (FinCo); a company managing and owning assets essential for the group’s business, including intellectual property, licences, know how, real estate (SPV); a company exercising managerial control over the group and acting as a group treasury as a result of a centralized cash management system (HoldCo); and a number of operational companies offering services or manufacturing products (OpCos). Administering such companies separately in insolvency can be difficult and suboptimal in terms of maximizing insolvency estate value, as the strict entity-by-entity treatment may lead to a breakup of intra-group links and a denial of access to vital resources and lifelines.

A good example is the Lehman Brothers group. When the holding company Lehman Brothers Holdings Inc. (LBHI) filed for Chapter 11 on 15 September 2008, many of its subsidiaries lost access to valuable sources of finance and information.^37^ The corporate legal shields, separating legal entities in the group, did not stop their failure. At least one of the reasons was that behind these shields, the entities were tied together in the web of debt and cross-guarantees. They depended on each other for the provision of financing, debt refinancing and services. For instance, the information on accounts and trades related to several group entities was frequently concentrated in one jurisdiction. Thus, Lehman Brothers International (Europe) (LBIE), the UK subsidiary of LBHI, recorded information on financial notes relevant to other group members, including its Dutch subsidiary, Lehman Brothers Treasury Co B.V. When the latter became insolvent in the Netherlands, the court needed such information to make asset distribution in the Dutch proceedings, as it was not readily available.^38^ A similar situation can arise if in insolvency entities lose their access to vital assets held by other entities in the group, including patents, licences, customer databases, real estate and raw materials. As a result, the operational activity of the group might be paralyzed, and group entities may end up in a piecemeal liquidation.

A group solution envisaged in the EIR Recast and the Model Law 2019 aims at preserving and maximizing the value of the insolvency estate for the benefit of creditors. It may also promote financial restructuring and rescue ailing companies or corporate groups. However, business rescue is usually incidental or subordinate to the goal of asset value maximization. In practice a group solution can be facilitated by the application of various tools. Among them (in the order of the rising coordination effect) are:

- Cooperation and communication between parallel insolvency proceedings. Importantly, the EIR Recast and the Model Law 2019 establish that insolvency practitioners and courts *shall* cooperate and communicate with each other to the maximum extent possible.^39^
- The conclusion of cross-border insolvency agreements or protocols.^40^ While the exact legal nature of insolvency protocols is not entirely clear (e.g. when it comes to their binding or non-binding character), since the 1990s they have played an increasingly important role in streamlining the administration of complex and large-scale cross-border insolvency cases. For example, they were used in such well-known cases as Lehman Brothers, Nortel Networks and Bernard Madoff Investment Securities. More recent examples of insolvency protocols concern airline insolvencies (e.g. Jet Airways, LATAM).
- The opening of special *sui generis* proceedings (i.e. group coordination proceedings under the EIR Recast and planning proceedings under the Model Law 2019).^41^ These special proceedings aim at improving coordination and simplifying information flows between parallel court proceedings, ultimately encouraging the development and implementation of a group insolvency solution.
- The appointment of the same insolvency practitioner in separate insolvency proceedings of group members.^42^ Such appointment greatly diminishes coordination problems, reduces transaction costs and may stimulate the preparation of a group‐wide strategy, including business rescue and the sale of an enterprise as a going concern.

### Limits of a Group Insolvency Solution in Corporate Insolvency Law

The optimal realization of the debtor’s assets is assessed on an entity-by-entity basis, as in the absence of substantive consolidation, the separateness of the asset pools of group entities is preserved. Any relation—by shareholding or otherwise—to another legal entity within a group usually becomes irrelevant. The well-known and widely used ‘best interests of creditors’ test (or the ‘no creditor worse off’ principle—NCWO principle),^43^ which allows creditors to veto the confirmation of a plan if they would receive less individually than in an alternative (typically, liquidation) scenario, is applied on the level of each separate company.

The ‘best interests of creditors’ test sets out a reference point or a minimum baseline, which shields individual creditors, guarantees fairness and ensures the protection of property rights.^44^ Thus, if a group reorganization plan or another group solution guarantees group survival and maximizes the value of the group as a whole (net group value) but harms the interests of creditors of a group member, such a plan cannot be confirmed, unless the affected creditors consent to it. In other words, as a general rule, the interests of a group or the majority of creditors cannot trump the interests of individual creditors.^45^ This limitation is also evident in the restrictions imposed on cross-border communication and cooperation, and the appointment of the same insolvency practitioner, discussed below.

The EIR Recast prescribes that in a group insolvency context courts and insolvency practitioners shall communicate and cooperate *to the extent that it does not entail any conflict of interest*.^46^ Such conflicts may arise in a situation of a dispute between group members and concern the enforcement of intra-group claims, transaction avoidance actions and the allocation or transfer of assets within the group.^47^ Moss and Smith note that whenever there is a disputed claim between two group entities, an obligation for insolvency practitioners to cooperate may ‘need to be circumscribed accordingly’.^48^ Schmidt provides another example of a conflict, when cooperation entails (gratuitous) ‘transmission of valuable know-how, patents, or business secrets, or making assets available to other group members which could have been disposed of with a large profit for the individual group member’.^49^ Conflicts of interest are magnified where the same insolvency practitioner is appointed to administer several members of an enterprise group with complex financial and business relationships and different groups of creditors.^50^ Taking into account the prominence of such conflicts, Van Galen writes that ‘[i]t is remarkable that there are many domestic cases in which the same liquidator is appointed in the insolvency proceedings of more than one group company’.^51^

The main idea behind restricting communication and cooperation or the appointment of a single insolvency practitioner in a situation of a conflict of interest is to minimize the potential harm that may otherwise be caused to the interests of individual group members (and their creditors), which maintain their separate legal identity. The restrictions are imposed to avoid the impairment of the value of one entity’s insolvency estate to the benefit of another group entity’s insolvency estate or the combined net group value.^52^

In sum, a group solution under the EIR Recast and the Model Law 2019 is a flexible concept that seeks to preserve group synergies and to safeguard and maximize the insolvency estate value to the advantage of creditors. It tries to solve the collective action problem triggered in insolvency by imposing group-level regulation and decision-making, aligning the economic self-interest of creditors with the goals of collective action on a group level and reducing cooperation costs and information asymmetries between separate proceedings.^53^ The achievement of a group solution may be facilitated by various legal tools, ranging from the simple exchange of information between insolvency practitioners and courts to the opening of special proceedings. The outer limits of a group solution are ultimately drawn up by the boundaries of a corporate form and the separateness of insolvency estates and pools of creditors, safeguarding creditors’ property rights and pre-insolvency entitlements.

## Group Solution and Group Resolvability in Bank Resolution

### Goals of Bank Resolution

The global financial crisis of 2008 (GFC) revealed the inadequacy of the then existing rules for the resolution of international (cross-border) credit institutions. The Basel Committee on Banking Supervision (BCBS) concluded in its report from 2010 that the ‘[e]xisting legal and regulatory arrangements are not generally designed to resolve problems in a financial group operating through multiple, separate legal entities’.^54^ This has resulted in the ‘predominance of the territorial approach in resolving banking crises and insolvencies’.^55^ A good example of this approach to the insolvency of a banking group is the case of Icelandic banks. Following the crisis of its outsized banking system in the autumn of 2008, the Icelandic government passed emergency legislation that granted protection to domestic deposits, which had been transferred to ‘new banks’, while foreign operations remained with the ‘old banks’, which were put into administration. Ultimately, foreign depositors received no support and had to be rescued by foreign (i.e. the UK and the Dutch) governments.^56^

The BRRD takes a different approach. It recognizes that the insolvency of a group entity can rapidly impact the financial soundness of the whole group^57^ and spread the contagion across the financial system, potentially causing a systemic crisis.^58^ As a result, various rules targeting banking groups and their resolution have been adopted in the EU, but also in non-EU countries (e.g. the USA).

The BRRD does not use the term ‘group solution’. Instead, it is concerned with and seeks to ensure group resolvability. Group resolvability means that a banking group should be allowed to fail in an orderly manner, without significant adverse consequences for the financial systems of the EU Member States in which group entities or branches are located, including broader financial instability or system-wide events, with a view to ensuring the continuity of critical functions carried out by those group entities.^59^ Thus, the focus of bank resolution is macroprudential, as it extends beyond the microprudential goal of asset value maximization and looks outside the boundaries of a single entity or an enterprise group.^60^ In addition to the interests of creditors, it takes other interests into account. Bank resolution seeks to preserve the stability of the financial system and to ensure the continuity of critical financial and economic functions performed by banks for the ultimate purpose of preventing social harm and public unrest. In this respect Lubben argues that the primary aim of bank resolution is not adjudicatory (i.e. collective claim enforcement), but regulatory.^61^

In light of the macroprudential purpose of bank resolution, the recognition of banking groups and a coordinated response to their financial distress appear logical, as the resolution of a single entity ignoring its interconnectedness and integration in the financial system may be short-sighted and hopelessly futile.^62^ The BCBS has demonstrated that the absence of a coordinated resolution mechanism for the legal entities in banking groups and financial conglomerates means that the only alternatives are often either a disorderly collapse (which may create systemic risks and lead to other disruptive consequences) or a bail-out.^63^

Group-level regulation in the context of bank supervision and bank resolution is especially evident in three spheres: (i) group recovery and resolution planning; (ii) group financial support agreements; and (iii) models of bank resolution and minimum requirements for own funds and eligible liabilities (MREL).

### Group Recovery and Resolution Planning

The BRRD establishes that it should be the general rule that ‘group recovery and resolution plans are prepared for the group as a whole and identify measures in relation to a parent institution as well as all individual subsidiaries that are part of a group’.^64^ The obligations for the adoption of recovery and resolution plans (colloquially referred to as ‘living wills’) have been introduced both in the USA^65^ and in the EU.^66^

There are several important reasons for drafting recovery and resolution plans. First, detailed and up-to-date plans provide resolution and other authorities with information, necessary for efficient monitoring, timely early intervention and the execution of resolution tools. Second, living wills play an educational and disciplining role and improve the awareness of the banks’ own management of the potential problems or weaknesses.^67^ As a result, they can serve as a warning indicator and a catalyst for action to remove impediments to the resolvability of an institution or a group. This may concern, inter alia, the enhancement of the corporate and financial structure of a bank or a banking group, its lines of business and organizational division of tasks between group entities. Third, living wills can contribute to the simplification of relations and the reduction of complexity within banking groups.^68^ Such complexity is common for banks and is frequently driven by extensive intra-group transactions.^69^ Fourth, ex ante crisis preparation and disclosure of (some) information in living wills may ‘clarify expectations and strengthen market confidence in the resolution actions of authorities’.^70^

The European Commission (EC) has adopted a regulation that specifies the content of recovery and resolution plans.^71^ For instance, as regards recovery plans, it clarified that such plans should be integrated in the overall corporate governance of the group and should provide for indicators or early warning signals, which may facilitate early action to remedy the unfolding crisis situation.^72^ Recovery plans also need to identify legal entities within the group in which core business lines and critical functions are located. The description of enterprise group members shall contain both the general characterization of the entities covered by the recovery plan and a detailed description of the group legal and financial structures, including intra-group exposures and funding relationships, such as intra-group guarantees, group financial support agreements and profit and loss transfer agreements.^73^

Thus, recovery plans generally cover the banking group globally and reveal its corporate, operational and legal interconnectedness. In light of such interconnectedness, recovery plans must include a list of all recovery options and describe each of them.^74^ Resolution plans should feature resolution strategies for each institution and group as a whole, highlight internal and external interdependencies which are critical to the maintenance of operational continuity, and outline the financing requirements and financing sources necessary for the implementation of the resolution strategy foreseen in the plan.^75^

### Group Financial Support Agreements

Intra-group financing between banking group members (between the parent and its foreign subsidiaries) constitutes a significant source of funds for cross-border operations.^76^ At the same time, financial distress may disturb the free flow of capital within banking groups, thereby impacting the financial soundness of the group and its constituent members. The BRRD acknowledges that the provision of financial support and assistance from one entity of a cross-border banking group to another entity in the same group may be restricted in a situation of financial distress.^77^ Such restrictions are usually designed to protect creditors and shareholders of each entity in the group. During the GFC, in order to protect domestic stakeholders many jurisdictions imposed or tightened restrictions on intra-group cross-border money transfers, limiting the ability of banking groups to optimally allocate liquidity.^78^

To address this problem, the BRRD has introduced a special regulatory regime, called a ‘group financial support agreement’, as a way to cope with the financial distress of subsidiaries in the recovery phase.^79^ The purpose of this regime is to ensure the predictability of flows of resources at different levels of a cross-border group in distress, to discourage local ring-fencing practices and to achieve the financial stability of a banking group without jeopardising the liquidity or solvency of entities extending financial support. Transactions falling under the protective regime of such agreements should be safeguarded from any legal impediments in national law,^80^ such as transaction avoidance rules.^81^

The EC has referred to a group support agreement as a ‘pre-emptive transaction’, highlighting that such a transaction is entered into pre-emptively and does not entail an immediate transfer of funds. It is rather a commitment or a promise to provide support, should the conditions for it be satisfied. Such support may take different forms, including the provision of a loan, a guarantee, assets to be used as collateral, or any combination of these forms of financial support.^82^ It can cover one or more entities in the group and involve support from the parent undertaking to subsidiaries (downstream), from subsidiaries to the parent undertaking (upstream) and between subsidiaries of the same group (cross-stream), or a combination of these variations.^83^

The macroprudential vision of bank resolution in the application of rules related to intra-group financial support materializes in the concept of a ‘group interest’. This is an innovation of the BRRD, which goes beyond a single-entity vision traditionally adopted in insolvency law. Under the BRRD, when approving the support agreement and the actual provision of financial support, competent authorities should analyse and compare the direct and indirect benefits for the group as a whole, which may result from rescuing an ailing group member. They should consider the potential risks for the group and the providing entity, created by the default and insolvency of the receiving entity. The group interest is also relevant for calculating the direct and indirect benefits for the grantor of financial support, arising from the restoration of the financial soundness of the receiving entity (i.e. group interest furthers entity interest). The European Banking Authority (EBA) accepts that such benefits might be difficult to quantify, for instance, when it comes to saving the reputation of the group.^84^

### Models of Bank Resolution and MREL

To ensure the internalization of losses in case of a bank failure and to prevent public bailouts, the BRRD has established rules on MREL. MREL should guarantee sufficient loss-absorbing and recapitalization capacity available in resolution, so that the costs of failure are borne by the bank’s investors, i.e. shareholders and creditors, instead of depositors or taxpayers. The internalization of losses is an essential pillar of the modern bank resolution framework. The requirement for global systemically important banks (G-SIBs) to have certain levels of loss absorption (i.e. bail-inable liabilities) has also been developed by the Financial Stability Board (FSB) and envisaged in the total loss absorbing capacity (TLAC).^85^

MREL and TLAC are closely tied to two main models or strategies of bank resolution: a single point of entry model (SPOE) and a multiple point of entry model (MPOE).^86^ The realization of these models requires a pre-positioning of the liabilities within the group.^87^ Under SPOE, resolution is undertaken at the level of a holding company placed at the top of the banking group (or sub-group).^88^ This strategy helps to avoid resolution actions and disruption at the level of group operating entities. In contrast, MPOE entails intervention by multiple resolution authorities and the application of resolution measures at the level of various operating companies or subsidiaries.^89^

In line with the TLAC standard, the BRRD recognizes both SPOE and MPOE resolution strategies. It also acknowledges the group reality^90^ and the need to ensure that loss-absorbing capacity is distributed across the banking group in accordance with the level of risk in its constituent group members and with reference to recovery and resolution plans.^91^ This may be done through the allocation of internal loss absorption and recapitalisation by upstreaming the losses from the subsidiaries to the ‘resolution entities’ (i.e. group entities to which resolution actions could be applied and to which rules on ‘external MREL’ apply).^92^ Such entities together with their subsidiaries comprise ‘resolution groups’. The group-internal distribution of MREL resources (referred to as ‘internal MREL’) should allow conversion and write down of debt without subsidiaries (non-resolution entities) themselves entering into resolution. Thus, financial distress is addressed at the group (or the sub-group) level rather than at an individual entity level.

Internal MREL and the concepts of ‘resolution entity’ and ‘resolution group’ are recent inventions and have been introduced in the EU by the so-called ‘banking package’, which, inter alia, includes the BRRD2.^93^ This new instrument requires resolution authorities to identity the resolution entities and resolution groups within banking groups and to appropriately consider the implications of any planned action within the group to ensure effective group resolution.^94^

### Limits of Group Resolvability

It is clear that the EU bank resolution regime recognizes that banks often operate as integrated and complex cross-border groups of companies. Therefore, it adjusts its approach to address group financial distress, inter alia, by way of the special tools discussed above (i.e. group recovery and resolution planning, group financial support agreements and models of bank resolution). This group-mindful regulation is much more comprehensive and developed compared to the rules applicable in the insolvency of non-financial companies, which mostly boil down to enhanced cooperation, communication and coordination. This divergence may stem from the specific policy goals pursued by (or the system of the values underlying) bank resolution. Unlike microprudential insolvency law with its primary focus on asset value maximization, bank resolution is concerned with the preservation of banks’ critical functions and national, regional and global financial stability. This macroprudential policy mandates the departure from a narrow entity-by-entity and territorial approach. Nevertheless, the extent of such a departure should not be overstated.

On closer examination, we can observe that the essence of entity separateness is diligently preserved in bank resolution.

First, the BRRD does not permit the application of resolution tools to a banking group as a whole, as if it is a single entity. Instead, resolution tools can only be applied at the level of separate group members (parent or subsidiary companies).^95^ This is without prejudice to the assessment of group resolvability. According to Article 2(1)(42) BRRD, ‘group resolution’ is comprised of either (i) the taking of resolution action at the level of a parent undertaking or of an institution subject to consolidated supervision (i.e. SPOE) with a view to resolving the whole or a part of the group, or (ii) the coordination of the application of resolution tools and the exercise of resolution powers by resolution authorities in relation to group entities that meet the conditions for resolution (i.e. MPOE).

Second, the NCWO principle remains the fundamental principle governing the protection of creditors’ rights in bank resolution.^96^ It guarantees that no creditor or shareholder shall incur greater losses in the course of the resolution action than it would have incurred if the bank had been wound up under normal insolvency proceedings.^97^ This principle is applied separately for each group entity. Thus, a resolution tool cannot be used if it worsens the position of a creditor compared to its position in a situation of normal insolvency proceedings, even if the resolution is in the public interest and increases the combined net value of the banking group.

The BRRD takes the national order of the priority of claims as a baseline, subject to the special rules, leading to a possible deviation from the national order of priorities.^98^ Thus, the reference point for comparison is national insolvency law. In the absence of a harmonized regime of insolvency laws, the NCWO principle would result in different outcomes across the EU in case of the resolution of a cross-border group. Should insolvency law provide for specific treatment for groups of companies (e.g. recognizing the existence of a group interest or providing for subordination of intragroup claims), this may affect the outcome of the NCWO assessment.^99^ Nevertheless, at this moment the vast majority of the EU Member States have not adopted tailored legislation addressing the insolvency of groups of companies or recognizing the group interest.

Third, the concept of a group interest evident in the application of the rules concerning group financial support agreements has serious limits. For example, the BRRD establishes that such agreements may not compromise the liquidity or solvency of providing entities as a result of granting rescue financing.^100^ In other words, the group interest should not trump the interests of individual group entities and their respective creditors. Besides, it appears that the BRRD does not introduce an overarching concept of a group interest, applicable across its provisions. Instead, a group interest surfaces only in the context of intra-group financial support.^101^

In sum, by a group solution in bank resolution, we may understand group resolvability. Unlike insolvency law, almost exclusively focused on optimal realisation of the debtor’s assets, bank resolution seeks to ensure a continuation of the critical functions of financial institutions and the preservation of financial stability. This macroprudential goal dictates the adoption of special group-mindful strategies and tools at the pre- and post-crisis stages. Among such tools are group recovery and resolution planning, group financial support agreements, pre-positioning of bail-inable liabilities and the realisation of SPOE or MPOE resolution strategies.^102^ Despite the macroprudential focus of bank resolution, the core of entity separateness remains untouched. This is evident in the entity-by-entity application of resolution tools and the NCWO principle, which may be considered an obstacle to a truly global group-wide bank resolution.

## Substantive Consolidation: An Ultimate Group Solution?

The previous sections described various approaches and strategies in dealing with corporate groups in financial distress. While signalling important developments towards the recognition of enterprise groups and addressing their peculiarities, they also embrace one key limitation. Group solutions proposed by the BRRD, EIR Recast, the Model Law 2019 and the national legal regimes respect entity separateness as a ground rule. Thus, the values underlying such instruments (i.e. the preservation of financial stability and estate value maximization respectively) do not outweigh the value of entity shielding that protects parties’ legitimate expectations, simplifies risk calculation and safeguards creditors’ property entitlements against specific legal entities.^103^ However, there is one important but rarely applied exception—the case of substantive consolidation.

Substantive consolidation leads to a pooling of assets and liabilities of several companies together, as if these companies constitute a single legal entity. As a result, the insolvency process becomes more simplified and cost-efficient, since there is only one procedure, one court, one insolvency estate and one insolvency practitioner. Substantive consolidation also eliminates cross-liability (e.g. intercompany contracts, group guarantees, joint liability, intercompany avoidance actions) for the simple reason that the separateness of legal entities and insolvency estates disappears. This makes intra-group litigation, which may last for years and consume large resources, unnecessary.

As efficient as it may be, substantive consolidation is not available in the majority of the EU Member States.^104^ The EIR Recast explicitly prohibits group coordination plans from including recommendations as to any consolidation of proceedings or insolvency estates.^105^ In those few jurisdictions where substantive consolidation is permitted, its application remains limited. It is typically restricted to cases of the intermingling of assets and liabilities within an enterprise group to a degree that ascertaining their actual ownership involves disproportionate expense or delay, or to cases of fraud and abuse of corporate form. For example, according to French law, the ‘commenced proceedings may be extended to one or more other persons where their assets are intermingled with those of the debtor or where the legal entity is a sham’.^106^ In a similar vein, Spanish law exceptionally allows substantive consolidation ‘when there is confusion of assets and it is not possible to separate the ownership of assets and liabilities without incurring an unjustified expense or delay’.^107^

Substantive consolidation is also possible in the USA but remains rare.^108^ The Federal Rules of Bankruptcy Procedure regulate consolidation or joint administration of cases against a debtor and an affiliate.^109^ However, this consolidation is procedural—insolvency cases of multiple debtors are routinely jointly administered by the US courts.^110^ Substantive consolidation is not distinctly authorized in the Bankruptcy Code and the majority of courts have based their decision to substantively consolidate on 11 U.S. Code §105, which provides that a court ‘may issue any order, process, or judgment that is necessary or appropriate to carry out the provisions’ of the Bankruptcy Code.

The power to order substantive consolidation was recognized by the US Supreme Court even before the enactment of the modern Bankruptcy Code as an equitable power.^111^ Different standards have since been employed by the courts.^112^ In assessing whether to authorize substantive consolidation they have taken into account: (i) whether creditors dealt with group entities as a single economic unit and did not rely on their separate identity; (ii) whether the affairs of the debtors are so entangled that consolidation will benefit all creditors;^113^ (iii) whether consolidation is necessary to avoid some harm or to realize some benefit; (iv) whether the demonstrated benefit of consolidation heavily outweigh the harm;^114^ (v) whether the assets and liabilities of the group entities are so scrambled that separating them is prohibitive and adversely affects all creditors.^115^

The extreme caution with which substantive consolidation is applied is premised on the fact that it affects the distribution between creditors, as creditors of a single debtor are forced to share the estate value with creditors of all consolidated entities. In the end, some creditors inevitably benefit at the expense of others. In other words, substantive consolidation leads to imposed wealth redistribution, profoundly affecting creditors’ (property) rights and recoveries. This is why the considerations of mere convenience or the maximization of the combined group insolvency estate are not sufficient to call substantive consolidation into play.

Disentangling the core value or the sole rationale underlying substantive consolidation is not easy, primarily because of the lack of a coherent theoretical framework and the variety of approaches taken in different jurisdictions (or even within a single jurisdiction, as is the case in the USA). On a very general level, substantive consolidation may be viewed as a consequence of the disregard or abuse of a corporate form.^116^ In such a context the boundaries between legal entities in the group become blurred and the group is perceived as a single economic and organizational unit—a consolidated legal entity.

If this is the case, we may inquire whether substantive consolidation can at all be regarded as a group solution. This is because such consolidation leads to the dissolution of the group or the death of the robust legal entity, which serves as a basis underpinning all other solutions explained above. It eliminates corporate boundaries and replaces a group as a system or a network of connected but distinct legal entities with a consolidated surviving entity. Can there be a group solution without a group? The answer should most likely be in the negative, at least to the extent that the legal separability is disregarded. From an economic perspective, the unity of an enterprise remains.

## Group Solution: Economic Rationale and Creditors’ Interests

### Pareto Efficiency v. Kaldor-Hicks efficiency

The previous sections have described the growing acceptance of the need to take a group environment into account when dealing with enterprise group insolvency. It was also stressed that despite the variety of tools to realize a group solution, one common feature restricting its scope is the protection of creditors of individual groups members. Both insolvency law and bank resolution establish the minimum safety net afforded to such creditors. This is particularly evident in the operation of the NCWO principle, limitations on communication and cooperation between insolvency practitioners and courts in a group insolvency context, restrictions related to the appointment of a single insolvency practitioner to administer several members of an enterprise group, and separateness in the application of the resolution tools.

Economically speaking, a group solution pursues a Pareto-efficient or Pareto-optimal solution to the extent that at least one party benefits from such a solution and nobody is made worse off.^117^ According to Morrison and Anderson, in insolvency the concept of Pareto efficiency is manifest ‘where an insolvency decision or choice produces a greater return to some creditors without reducing the return to any other creditor’.^118^ In a group insolvency context, Pareto efficiency can, for instance, be achieved by producing greater returns to (some) creditors, ensuring the survival of group entities (and thus, preserving employment), safeguarding financial stability and banks’ critical functions and reaching other substantive goals, without at the same time damaging the interests of (non-consenting) creditors, whose position is compared to a baseline scenario (i.e. a no-group-solution scenario). As discussed above, this is generally done through addressing the collective action problem at the group level—we can call it a group-mindful creditors’ bargain.

In law, Pareto efficiency prevails over another type of economic efficiency—Kaldor–Hicks efficiency. Kaldor–Hicks efficiency approves an outcome that maximizes a net gain (total wealth maximization), even if potentially leaving some parties worse off. For example, Kaldor–Hicks efficiency will be reached if the aggregate returns to creditors increase, while some creditors have to suffer, provided that the benefits to those creditors who are better off exceed the losses of creditors who are worse off. This outcome is normatively unattractive since it may encroach on creditors’ property rights, pre-insolvency entitlements and legitimate expectations. Of course, those who ‘win’ could in principle and theoretically compensate those who ‘lose’, but this is not required for Kaldor–Hicks efficiency.^119^

### Determining ‘Better Off’ and ‘Worse Off’

The determination of the principle of economic efficiency underlying a group solution does not by itself clarify what it means for a creditor to be ‘worse off’ or ‘better off’. Clarification of these terms and their flexible and multifaceted interpretation is key to the effective implementation of group solutions and, more generally, to the proper operation of rules dealing with group insolvencies. This should start with the establishment of the ‘interest’ assigned to creditors. In my opinion, such an interest should not be limited to a myopic focus on short-term profits. Instead, a more balanced approach, comprehensively looking at the medium and long-term direct and indirect benefits of a group solution should be embraced. Admittedly, a determination of such benefits may sometimes be problematic and give rise to litigation. The discussion of the topic of creditors’ interest is reminiscent of the debates around the concept of shareholder primacy, corporate purpose and the problem of short-termism in company law.^120^ This makes sense, as in insolvency creditors become residual claimants or economic owners of the company, ultimately replacing shareholders.^121^

A group solution might cause some (monetary) losses to some creditors in the short term, but directly and indirectly benefit them and other stakeholders in the mid or long term, finally approaching Pareto efficiency. For instance, a group solution, even if resulting in a short-term detriment, may preserve the running enterprise and allow creditors to continue contracting and benefiting from the ongoing relationships and future income. A determination of ‘benefit’ and ‘interest’ should therefore be carried out against the background of the group reality. This approach has been embraced by UNCITRAL, the World Bank and the BRRD in the rules and recommendations concerning intra-group rescue financing.

The World Bank stipulates that the insolvency system should ‘permit an enterprise group member subject to insolvency proceedings to provide or facilitate post-commencement finance or other kind of financial assistance to other enterprises in the group which are also subject to insolvency proceedings’.^122^ Similarly, UNCITRAL accepts that insolvency law should permit an enterprise group member subject to insolvency proceedings to advance post-commencement finance or grant a security interest/provide a guarantee to another enterprise group member subject to insolvency proceedings.^123^ As a result, some detriment, even if only in the short term, to the interests of an individual group member and its creditors for the long-term benefit of the enterprise may be justified.^124^ The same logic is adopted in the BRRD’s provisions concerning intra-group financial support agreements, as discussed above. The BRRD adheres to a nuanced approach in defining the interest of the group entity providing support to another (financially distressed) group entity. When identifying the interests of the providing entity and its creditors, it accounts for direct and indirect benefits for such an entity, including those resulting from a recovery of the group as a whole, as well as the risks that would result from the destabilisation of the group.^125^

The group-sensitive and forward-looking interpretation of creditors’ interest may facilitate commercially sensible and practical group solutions, going beyond the short-term satisfaction of creditors’ basic (minimum) entitlements.

## Group Solution as a Principle of Insolvency Law

Insolvency law has made significant progress in the last few decades. This progress has been evidenced in the increasing harmonization of private international law rules and, somewhat more modestly, in substantive insolvency law and bank resolution. The harmonization was driven by the recognition and acceptance of certain legal principles. Such principles can be defined as ‘fundamental and basic standards’^126^ or ‘meta-norms’.^127^ As opposed to rules and policies, principles have a higher level of abstraction and are arguably more stable. To be accepted as a principle, a standard must be widely and lastingly recognized.^128^ It can be implemented in a variety of rules, often in different areas of the law. Nevertheless, the social and economic background affects the importance assigned to or the relative weight of a legal principle, or its position in case of a conflict with another principle.^129^ It may also cause new principles to appear and supplement or substitute existing ones. Thus, legal principles are not set in stone and develop over time.

The modernization of insolvency law has resulted in the appearance of various guidelines and rules for the insolvency of enterprise groups. They have been introduced or proposed at national (e.g. Germany^130^), regional (e.g. EIR Recast and BRRD) and global (e.g. Model Law 2019, FSB’s Key Attributes) levels. The question arises whether based on these developments it is possible to contemplate that a new stand-alone principle of insolvency law—a group solution—has taken shape. Based on the analysis in the preceding sections of this article, I conclude that such a principle has not (yet) crystallized, but this may change as the group reality keeps on being recognized and acted upon by insolvency/restructuring law. This slow crystallization process may be explained by the fact that there is no single concept or understanding of a group solution. The inherent flexibility and adaptability of a group solution is both its strength and its weakness.

The main strength of a group solution is the capability to fit into the specific circumstances and broader context (e.g. properties of a corporate group, the nature of its business, characteristics of financial distress, the policy goals pursued).^131^ For example, a group solution may come down to the coordination of parallel insolvency or restructuring proceedings for the purpose of preparing and executing a group reorganization plan (i.e. a combination of separate reorganization plans). It might also entail the improvement of group resolvability by mandating the adoption of group recovery and resolution plans and the application of resolution measures to resolve a failing banking group. In its application, a group solution seems to promote different policy objectives with distinct underlying values. Under the EIR Recast and the Model Law 2019, a group solution aims at maintaining group synergies and operational continuity in order to maximize the value of the overall insolvency estate. The BRRD stresses group resolvability, which is primarily concerned with preserving the systemically important activities of credit institutions and with ensuring financial stability.

At the same time, the flexibility and sometimes ambiguous character of a group solution may be its weakness and explain the frequent lack of action to address group financial distress, the variety of approaches to treating enterprise groups in insolvency and the slow process of legal harmonization in this area. It appears that a group solution does not have an independent policy goal of its own. Instead, it plays a crucial but supporting role and aims at promoting the foundational principles of insolvency law, such as the principles of the maximization of the value of a firm’s assets and recoveries by creditors, the universality of insolvency proceedings (as applied in a group context), and a predictable and efficient insolvency process.^132^ It is also integral to effective bank resolution, which seeks to guarantee the continuity of critical functions (e.g. payment, clearing and settlement), to protect public funds and to avoid adverse effects on financial stability.^133^

However, the importance of a group solution should not be underestimated. It can emphasize the similarities of practices utilized in different jurisdictions and legal instruments (instead of highlighting the differences) and sharpen our understanding of the principles and goals of insolvency law and bank resolution. It might also facilitate a departure from the path-dependent entity-by-entity administration of group insolvencies and the development of new harmonized tools and practices to tackle problems common to group insolvency at national, regional and global levels. A group solution and legal rules promoting it may signal a victory for economic substance and business reality over legal formalism.

## Conclusion

The concept of a group solution is relatively new and is still developing. This article has traced the emergence of this concept and its different manifestations in various legal regimes. A group solution was caused by the recognition of the specificity of problems related to the insolvency of enterprise groups. This has not happened at once, but has resulted from the evolution of views and ideas, evident in hard and soft law instruments of the 2000s and the 2010s. Corporate groups are explicitly acknowledged in the BRRD (2014), the EIR Recast (2015) and the UNCITRAL Model Law on Enterprise Group Insolvency (2019). Special rules dealing with the insolvency of enterprise groups have also found their way into national law (e.g. Germany).

This article has explored the concept of a group solution by looking at five questions, raised in the introduction, namely: (i) what is a group solution?; (ii) which goal does a group solution seek to achieve?; (iii) how does it try to achieve it?; (iv) what are the limitations or boundaries of a group solution?; and (v) what are the strengths and weaknesses of modern approaches to a group solution?

Based on the analysis of modern legal instruments and practices in corporate insolvency and bank resolution, I have come to the conclusion that despite the gradual acceptance of the group phenomenon, a group solution has not yet formed as a coherent and well-defined legal principle with its own substance and distinct purpose. Instead, it represents a series of various approaches, tools and practices addressing problems that are characteristic of enterprise groups. On the one hand, this variability guarantees flexibility, corresponding to the diversity of organizational and financial structures of corporate groups, their varying levels of integration and managerial centralisation. On the other hand, it could hinder harmonization attempts and the development of group solutions across national borders. Further complication comes from the fact that a group solution, or to be more precise, group solutions, pursue different policy objectives underpinned by different societal values.

First, in corporate insolvency a group solution seeks to facilitate asset value maximization through active communication and cooperation, realized by way of cross-border insolvency protocols, special coordination or planning proceedings and the appointment of a single insolvency practitioner. In this respect, communication and cooperation act as a precondition for the implementation of more substantive group solutions (e.g. the development of a group reorganization plan safeguarding the operational continuity and going concern value of the business). The latter strives to solve the collective action problem at a group level and therefore prevents group disintegration or a diminution of its value over time. It attempts to create a structured bargaining process, bringing order to the otherwise complex and atomized insolvency process. Consequently, it brings down coordination and motivation costs.^134^

Second, in bank resolution a group solution pursues the goal of protecting society from systemic risks and ensuring the continuity of critical services and avoiding adverse effects on financial stability. This is, for instance, achieved by means of special strategies in the field of bank resolution. Such strategies embrace a proactive and precautionary approach and include group recovery and resolution planning, group financial support agreements and the pre-positioning of liabilities within a group with a view to a particular resolution arrangement (MPOE or SPOE). It appears that a group solution and its tools in the area of bank resolution are more advanced and far-reaching compared to those adopted in corporate insolvency. This is the result of the macroprudential vision increasingly embraced in the wake of the GFC.

Third, in exceptional circumstances the disregard or the abuse of a corporate form might sanction substantive consolidation, which treats separate legal entities as if they are merged into a single entity with cumulative assets and liabilities. The doctrine of substantive consolidation is recognized in the USA, France and Spain, but its underlying rationale and the conditions for its application diverge. Strictly speaking, substantive consolidation should not be considered a group solution, because as a consequence the group of companies as a system or a network of distinct legal entities with separate pools of assets and groups of creditors ceases to exist. I claim that in the absence of a group, there can hardly be a group solution.

Group solutions proposed by various legal instruments discussed in this article have one important limitation. A group solution cannot trump the interests of individual group members and their creditors. This is based on the premise that group entities retain their separateness in insolvency and that creditors’ claims are protected by property law. This protection is realized through the operation of the NCWO principle, restrictions aimed at minimizing the risks and negative effects of conflicts of interest in group insolvency scenarios and the entity-by-entity application of resolution measures in the context of banking group resolution.

Nevertheless, various limitations, generally sought to preserve the merits of corporate separateness, should not bar Pareto-efficient group solutions, which help to achieve the goals of insolvency law and bank resolution and operationalize them to a greater extent that would ever be possible without such group solutions (i.e. the no-group-solution scenario). To make Pareto-efficient group solutions possible and realize their full potential, I have argued that it is necessary to rethink how we define creditors’ ‘interest’ and to consider both medium and long-term direct and indirect benefits for creditors, as well as a broader enterprise group and social environment, instead of solely focusing on short-term direct monetary satisfaction of creditors’ claims.

## Footnotes

1 For an overview of insolvency law responses to COVID-19, see The COVID-19 Pandemic and Business Law: A Series of Posts from the Oxford Business Law Blog, Oxford Legal Studies Research Paper No. 15/2020 (April 15, 2020). See also Gurrea-Martínez (2020).

2 Capri (2020).

3 Adler (2018), p 1855.

4 Mevorach (2009), p 153.

5 Regulation (EU) 2015/848 of the European Parliament and of the Council of 20 May 2015 on insolvency proceedings [2015] OJ L 141/19 (EIR Recast).

6 UNCITRAL Model Law on Enterprise Group Insolvency (2019) (Model Law 2019).

7 Directive 2014/59/EU of the European Parliament and of the Council of 15 May 2014 establishing a framework for the recovery and resolution of credit institutions and investment firms [2014] OJ L 173/190 (BRRD).

8 The Model Law 1997 seeks to offer ‘effective mechanisms for dealing with cases of cross-border insolvency’ and as of June 2021 has been adopted in 49 States in a total of 53 jurisdictions.

9 Directive 2001/24/EC of the European Parliament and of the Council of 4 April 2001 on the reorganisation and winding up of credit institutions [2001] OJ L 125.

10 Council Regulation (EC) No. 1346/2000 on insolvency proceedings [2000] OJ L 160/1. The Virgos–Schmit Report (1996), supplementing the European Insolvency Convention (1995), the predecessor of the original European Insolvency Regulation, directly stated that the ‘Convention offers no rule for groups of affiliated companies (parent-subsidiary schemes)’.

11 UNCITRAL Working Group V, Thirty-eighth session, UNCITRAL Legislative Guide on Insolvency Law, Part three: Treatment of enterprise groups in insolvency, 11 February 2010, p 3.

12 See Teichmann (2016), p 150, pointing out that ‘the company law of most jurisdictions is still focussed on the single legal entity and does not expressly acknowledge the fact that the corporate group may create legal challenges which are different from those of an independent company’.

13 Hansmann et al. (2006), p 1338 noting that ‘entity shielding is the *sine qua non* of the legal entity’.

14 Wessels and Madaus (2017), para. 697.

15 Hopt (2018), p 612.

16 Dammann (2019), p 231.

17 For instance, in the UK the suspect period for preference transactions is 2 years for connected persons, compared to the otherwise applicable 6-month period, see section 240, UK Insolvency Act 1986. In the USA, the preference period is extended from the usual 90 days to 1 year in case of an insider (11 U.S. Code § 547).

18 According to § 131(2) German Insolvency Code (InsO), a person with a close relationship to the debtor on the date of [preference] transaction (§ 138) shall be presumed to have been aware of the disadvantage to the creditors in insolvency proceedings. Under section 239(6) UK Insolvency Act 1986, a company which has given a preference to a person connected with the company ‘is presumed […] to have been influenced in deciding to give it by such a desire as is mentioned in subsection (5)’ [a desire to create an advantage for a counterparty]. In Greece, avoidance of preferences requires knowledge of the counterparty about the detrimental effects of a transaction. Such knowledge is presumed for connected persons, see Art. 44(2) Greek Bankruptcy Code. Knowledge of prejudice in related-party transactions is codified in Art. 43 Dutch Bankruptcy Act.

19 Art. 228 Spanish Insolvency Act.

20 A group of scholars, Forum Europaeum Corporate Group Law, has recommended the introduction of a modified *Rozenblum* doctrine at the European level. See Corporate Group Law for Europe, *EBOR* 2000, pp 165–264. See also Report of the High-Level Group of Company Law Experts on Model Regulatory Framework for Company Law in Europe (‘Winter Report’) (2002).

21 EC, Action Plan: European company law and corporate governance—a modern legal framework for more engaged shareholders and sustainable companies, COM/2012/0740 final.

22 Conac (2013); Winner (2016).

23 Posner (1976).

24 See Hansmann and Squire (2018), p 259, arguing that corporate partitioning does not generate the many benefits often attributed to it.

25 The dichotomy between a ‘single entity approach’ and an ‘enterprise approach’ has been noted in the UNCITRAL Legislative Guide on Insolvency Law, Part three (2010).

26 See UNCITRAL Legislative Guide on Insolvency Law, Part three, Chapter II, paras. 105–137.

27 For example, according to Art. 2(1)(26) BRRD, a ‘group’ means a parent undertaking and its subsidiaries. A similar definition can be found in Art. 2(13) EIR Recast. The Model Law 2019 in Art. 2(b) defines an ‘enterprise group’ as two or more enterprises that are interconnected by control or significant ownership.

28 *Gesetz zur Erleichterung der Bewältigung von Konzerninsolvenzen, 13.04.2017, Bundesgesetzblatt Jahrgang 2017 Teil I Nr. 22, ausgegeben am 21.04.2017*, p 866.

29 Madaus (2019), §15.04. For the country-by-country study of approaches to company groups, see also Manóvil (2020).

30 A scheme of arrangement is a statutory procedure available under Part 26 (sections 895-901) of the UK Companies Act 2006. It is not considered to be an insolvency procedure, as it falls under company rather than insolvency law. Nevertheless, in practice schemes are used by companies (and corporate groups) in distress as a mechanism for financial reorganization. For recent examples, where the schemes have been employed to prevent group failures, see *DTEK Energy BV, Re* [2021] EWHC 1551 (Ch), citing among other factors, the evidence that if ‘the schemes are not implemented it is “highly likely” that disordered “domino” insolvencies would occur across the Group’. *Re Codere Finance 2 (UK) Limited)* [2020] EWHC 2683 (Ch), noting that ‘object of the Scheme is to compromise the claims of Scheme creditors against all obligors in respect of the Existing Notes, including the co-issuer (Codere Finance) and Group guarantors.’

31 Payne (2014), p 24. Third-party releases are also prevalent in Ireland, Singapore and Australia. In the case of *In the matter of Nordic Aviation Capital Designated Activity Company* [2020 No. 162 COS.], the High Court of Ireland cited with approval the judgment of the Court of Appeal in Singapore in *Pathfinder Strategic Credit LP v. Empire Capital Resources PTE Ltd.* [2019] SGCA 29, which held that the jurisdictional test for third-party releases should be applied ‘in a commercially sensible manner particularly where a group restructuring is concerned’. On third-party releases, read further Kokorin (2021a).

32 *In the matter of Syncreon Group BV* [2019] EWHC 2412 (Ch), 2019 WL 04279919. Third-party releases are commonly approved with respect to group members of (foreign) companies. See *In Re La Seda de Barcelona SA* [2010] EWHC 1364 (Ch); *Re Magyar Telecom BV* [2013] EWHC 3800 (Ch); *In the Matter of New World Resources N.V.* [2014] EWHC 3143 (Ch); *Re NN2 Newco Limited* [2019] EWHC 1917 (Ch), holding that the ‘likely alternative to the capital restructuring which has emerged is group insolvency in multiple jurisdictions: that effectively means liquidation in one form or another because of the absence of restructuring procedures in other jurisdictions.’

33 EIR Recast, Recital 52.

34 The Model Law 2019 was approved by UNCITRAL on 15 July 2019. As of June 2021, it has not been transposed at the national level.

35 Model Law 2019, Art. 2(g).

36 Guide to Enactment of the UNCITRAL Model Law on Enterprise Group Insolvency (Guide to Model Law 2019), para. 42.

37 See Lehman Brothers Holdings Inc. Chapter 11 Proceedings Examiner’s Report (Valukas Report) (2010), p 1550, describing Lehman’s centralized intra-group cash pooling system. McDonald (2015), p 117, explaining the practical issues as each subsidiary was cut off from the others and LBHI, so that ‘[i]t was not possible to generate information and liquidate assets efficiently and identify ways to maximize value.’ Just how complex the situation was can be derived from the words of Lehman’s lawyer Harvey Miller, who reported that ‘[a]ll the accumulated information in Lehman’s systems totals 2000 terabytes of data, an amount that would completely fill 20,000 computers to the maximum. This vast sea of information spreads across 2700 software systems applications and is dispersed throughout ledger accounts in the numerous subsidiaries […]. The financial information must be retrieved […] and collated and cross-referenced for accuracy and consistency.’ See Testimony of Harvey R. Miller before the Subcommittee on Commercial and Administrative Law of the House of Representatives Committee of the Judiciary, 111th Congress, 1st Session for Hearings on ‘Too Big to Fail: The Role of Bankruptcy and Antitrust Law in Financial Regulatory Reform’, 22 October 2009.

38 Kirshner (2018), p 5, noting that uncoordinated insolvency proceedings faced the problem of separating intercompany arrangements, representing an elaborate system of cross-collateralization and intercompany guarantees.

39 EIR Recast, Arts. 56-58; Model Law 2019, Arts. 9, 14.

40 EIR Recast, Art. 56; Model Law 2019, Art. 16. On cross-border insolvency protocols, see Kokorin and Wessels (2021).

41 For a discussion and comparison of these proceedings, see Mevorach (2019).

42 EIR Recast, Recital 53; Model Law 2019, Art. 17.

43 11 U.S. Code § 1129(a)(7). On the US origins of this principle, see Tabb (1995). Since then, the ‘best interests of creditors’ test has entered into the national laws of many European countries. See e.g. § 251(1) InsO (Germany), Arts. 153(2)(1), 272(2)(1) and 338(2) Dutch Bankruptcy Act. This test is also mandated by Directive (EU) 2019/1023 of 20 June 2019 on preventive restructuring frameworks, on discharge of debt and disqualifications, and on measures to increase the efficiency of procedures concerning restructuring, insolvency and discharge of debt [2019] OJ L 172/18 (Restructuring Directive). Under the latter, satisfying the ‘best-interest-of-creditors should be considered to mean that no dissenting creditor is worse off under a restructuring plan than it would be either in the case of liquidation, whether piecemeal liquidation or sale of the business as a going concern, or in the event of the *next-best-alternative scenario* if the restructuring plan were not to be confirmed’. See Recital 52, Arts. 1(6), 10(2)(d). Read further Wessels and Madaus (2020), pp 288–289. See also Krohn (2021).

44 Madaus (2018), p 638.

45 See also UNCITRAL Legislative Guide on Insolvency Law, Part four: Directors’ obligations in the period approaching insolvency (including in enterprise groups) (2019), Section two, para. 8, noting that typically ‘collective benefit is not a sufficient justification by itself for acts judged to be prejudicial to creditors’.

46 EIR Recast, Arts. 56-58.

47 See UNCITRAL Legislative Guide, Part three, Chapter II, para. 68, referring to a situation of intra-group rescue financing where a ‘conflict of interest might arise, for example, in balancing the interests of the group as a whole against the potentially different interests of the lender and the receiver of post-commencement finance’.

48 Moss and Smith (2016), para. 8.754.

49 Schmidt (2016), para. 56.22.

50 Guide to Model Law 2019, para. 103.

51 Van Galen (2012), p 36. He also points out that ‘conflicts of interests between individual group companies may be much more pronounced in insolvency proceedings’.

52 On the issue of conflicts of interest and the procedural coordination of group insolvencies, see Kokorin (2020a).

53 For a description of the collective action problem, see Jackson (1986), pp 16-17, viewing the role of insolvency law as one of ‘ameliorating a common pool problem created by a system of individual creditor remedies’.

54 BCBS, Report and Recommendations of the Cross-border Bank Resolution Group (March 2010), p 25.

55 Ibid., p 4.

56 For discussion of this and other cases of poor cross-border cooperation in banking crises, see Schoenmaker (2013), Ch. 4.2. See also Baudino et al. (2020).

57 BRRD, Recital 11.

58 BRRD, Art. 2(30), defining ‘systemic crisis’ as ‘a disruption in the financial system with the potential to have serious negative consequences for the internal market and the real economy’.

59 BRRD, Art. 16.

60 On the distinction between micro- and macro-prudential functions of insolvency law and bank resolution, see Kokorin (2021b).

61 Lubben (2018), p 1396.

62 Ringe and Patel (2019), explaining why the application of bail-in resolution powers, disregarding the growing interconnectedness of European banks, may actually further systemic risk.

63 BCBS, Report and Recommendations of the Cross-border Bank Resolution Group (March 2010), p 24.

64 BRRD, Recital 33.

65 Dodd-Frank Act, section 165(d). The US legislation does not separate recovery and resolution planning.

66 BRRD, Arts. 5 and 10. Under the BRRD, recovery plans are drawn up by the banks themselves, while resolution plans are prepared by the Single Resolution Board and national resolution authorities. In contrast, under the Dodd-Frank Act, banks must draft resolution plans and submit them to the Federal Reserve Board, the Financial Stability Oversight Council and the Federal Deposit Insurance Corporation.

67 Ventoruzzo and Sandrelli (2019), p 20.

68 Avgouleas et al. (2013), p 211.

69 Binder (2014), p 6.

70 FSB, Public Disclosure on Resolution Planning and Resolvability. Discussion Paper for Public Consultation (June 3, 2019). It should be noted that in the EU recovery and resolution plans are not publicly available. However, some information therein must be revealed (e.g. general terms of group financial support agreements, see Art. 26 BRRD). In the US resolution plans consist of a private section that contains confidential supervisory and proprietary information that is not available to the public, and an open public section.

71 Commission Delegated Regulation (EU) 2016/1075 of 23 March 2016 supplementing Directive 2014/59/EU of the European Parliament and of the Council with regard to regulatory technical standards specifying the content of recovery plans, resolution plans and group resolution plans, the minimum criteria that the competent authority is to assess as regards recovery plans and group recovery plans, the conditions for group financial support, the requirements for independent valuers, the contractual recognition of write-down and conversion powers, the procedures and contents of notification requirements and of notice of suspension and the operational functioning of the resolution colleges [2016] OJ L 184/1.

72 See EBA, Guidelines on the minimum list of qualitative and quantitative recovery plan indicators, Final report, EBA-GL-2015-02 (May 6, 2015).

73 Commission Delegated Regulation (EU) 2016/1075, Art. 7. See also EBA, Recommendation on the coverage of entities in a group recovery plan, Final report, EBA/Rec/2017/02 (November 1, 2017).

74 Commission Delegated Regulation (EU) 2016/1075, Art. 8.

75 Ibid., Art. 22. On interlinkages between recovery and resolution plans, see EBA, Report on Interlinkages between Recovery and Resolution Planning, EBA/Rep/2020/16 (May 20, 2020).

76 Reinhardt and Riddiough (2015). See also De Haas and Van Lelyveld (2010), showing how multinational banks form their own internal capital markets, managing the credit growth of their subsidiaries.

77 BRRD, Recital 38.

78 Cerutti et al. (2010), p 5. See EC, Final Report, Study on the feasibility of reducing obstacles to the transfer of assets within a cross border banking group during a financial crisis (2010).

79 For a discussion of group financial support agreements, see Kokorin (2020b). See also Gardella et al. (2020), acknowledging the utility of intra-group financial support agreements (calling them ‘one of the most important existing legal/regulatory devices to govern home and host authorities’ diverging interests and prerogatives in cases of financial distress’), but criticizing the excessive rigidity of their regulation.

80 BRRD, Art. 19(4).

81 Subordination rules, found in some national insolvency laws, can also be treated as ‘legal impediments’ in the meaning of Art. 19(4) BRRD.

82 BRRD, Art. 19(5)(b).

83 BRRD, Art. 19(5)(a).

84 EBA, Guidelines specifying the conditions for group financial support under Art. 23 of Directive 2014/59/EU, 9 July 2015, EBA/GL/2015/17.

85 FSB, Principles on Loss-absorbing and Recapitalisation Capacity of G-SIBs in Resolution. Total Loss-absorbing Capacity (TLAC) Term Sheet (2015).

86 Davies (2015), p 262, distinguishing between ‘multiple point of entry without coordination’ and ‘multiple point of entry with a high level of coordination’. See also Bolton and Oehmke (2019), p 2415, highlighting the need for complementarity between a bank resolution strategy and the structure of global banks.

87 On the pros and cons of pre-positioning, see Quarles (2019).

88 SPOE strategy is adopted by JPMorgan Chase, see JPMorgan Chase, Resolution Plan Public Filing (2019). This resolution plan notes that SPOE strategy ‘would limit the destabilizing effects of a possible failure by avoiding bankruptcy for the firm’s subsidiaries’.

89 MPOE strategy is embraced by Banco Santander SA, see Banco Santander SA, Resolution Plan for U.S. Operations: Public section (December 31, 2018). The Resolution Plan states that ‘the Group’s organizational structure permits clear and precise distinction between the main business units. It also makes it possible to separate particular units from the rest of the Group if the intention were to dispose of any particular unit or should it be necessary to isolate any unit in the case of a resolution scenario’. This structure, according to the resolution plan, ‘makes the “multiple points of entry” the most appropriate resolution strategy for the Santander Group’.

90 Some may argue that a MPOE resolution strategy does not represent a group solution, since the banking group is resolved entity-by-entity, through individual resolution of each institution within the group. However, MPOE strategy may be designed to better reflect the organizational, financial, geographical and strategic characteristics of a particular banking group. For example, in its resolution plan, HSBC Group states that its resolution is ‘facilitated by the separability embedded within the structure of the HSBC Group’. As a result, the preferred resolution strategy is based on the ‘resolution and restructuring of regional or national groups of affiliated companies’. HSBC Holdings Plc, SIFI Plan, Section I—Public Section (2018).

91 BRRD, Recital 80.

92 External MREL refers to debt instruments issued by a resolution entity to third parties. In case of a bail-in, these third parties will suffer losses. In contrast, internal MREL instruments are issued by group entities that are not themselves resolution entities. Such instruments are issued to a group resolution entity.

93 Directive (EU) 2019/879 of the European Parliament and of the Council of 20 May 2019 amending Directive 2014/59/EU as regards the loss-absorbing and recapitalisation capacity of credit institutions and investment firms and Directive 98/26/EC [2019] OJ L 150/296.

94 BRRD2, Recital 4.

95 Deslandes and Magnus (2018).

96 BRRD, Art. 34(1). The NCWO principle aims at protecting the right to property and preventing illegal expropriation. See De Serière and Van der Houwen (2016), p 377. See also Martino (2020), emphasizing that the policy goals of preserving financial stability and protecting property rights (i.e. by way of the NCWO rule) clearly outweigh other considerations, including the minimization of resolution costs or the avoidance of value destruction. For criticism of the NCWO principle, see Schillig (2020), p 179, asserting that the NCWO calculation is vague and essentially ex post. See also Singh (2020), pp 137–138, underscoring that the NCWO principle ‘provides a balance between the competing public and private interests’, but recognising that in a group context ‘it is likely that the [NCWO principle] will lead to different outcomes for the creditors of the different incorporated entities’.

97 BRRD, Arts. 73-75. A breach of the NCWO principle gives rise to the compensation claim by the relevant affected creditors and shareholders.

98 BRRD, Art. 108.

99 EBA, Single Rulebook Q&A, NCWO principle in a group resolution, Question ID 2015_2458.

100 BRRD, Art. 23(1)(e).

101 Ferran and Ho (2014), p 41.

102 There are other mechanisms aiming at group-mindful bank resolution. See e.g. Art. 68 BRRD, neutralizing cross-entity ipso facto clauses and Art. 88 BRRD, mandating cooperation and consultation in resolution colleges. The achievement of a group solution is also promoted by a significant level of centralisation in the decision-making within the Banking Union.

103 According to Posner, corporate division and limited liability aim at solving the problems of information and supervision. Instead of appraising the enterprise in its entirety, the creditors only need to look into the existing and expected assets and liabilities of a separate legal entity, which should arguably be cheaper. Posner (1975). In practice, however, the clear-cut division of an enterprise into the isolated islands of assets and liabilities does not occur. Ayotte and Hansmann note that the ‘ambiguity of entity boundaries, therefore, may in fact raise creditor-monitoring costs in large corporate groups, not lower them’. Ayotte and Hansmann (2013), p 722.

104 For a concise overview of European approaches, see Wessels and Madaus (2017), pp 348–349. See also Sax et al. (2018). For the criticism of the lenient approach to substantive consolidation in Brazil, see Neder Cerezetti (2021).

105 EIR Recast, Art. 72(3).

106 Art. L. 621-2 Commercial Code.

107 Art. 43 Spanish Insolvency Act. Notably, this provision introduces purely objective criteria, which do not consider fraudulent or abusive intentions by debtors. Read further in Naharro (2020).

108 Graulich (2006), p 528, emphasizing that ‘every court of appeals to consider the issue has held that substantive consolidation is an “extraordinary” remedy that should rarely be invoked’.

109 Fed. R. Bankr. P. 1015(b).

110 Levitin (2019), p 661.

111 *Sampsell v. Imperial Paper & Color Corporation*, 313 U.S. 215 (U.S. Sup. Ct. 1941).

112 For an overview, see Bowling (2010). Also Widen (2008).

113 *In re Augie/Restivo Baking Co.*, 860 F.2d 515 (2d Cir. 1988).

114 *Drabkin v. Midland-Ross Corp. (In re Auto-Train Corp.)*, 810 F.2d 270, 276 (D.C. Cir. 1987).

115 *In re Owens Corning*, 419 F.3d 195, Bankr. L. Rep. P 80, 343 (U.S. C.A. 3rd Cir. 2005).

116 See Westbrook (2018), p 36, supporting the regime that ignores the corporate form when it has been carelessly or deliberately ignored by the management of the group.

117 Cooter (1987), p 151.

118 Morrison and Anderson (2013), p 196.

119 Posner (2010), p 17. If such compensation is secured, the outcome could be similar to the Pareto-efficient outcome, since the detriment caused by a group solution to some creditors is remedied. This is why Kaldor-Hicks efficiency is sometimes termed the ‘potential’ Pareto efficiency.

120 See Bebchuk and Tallarita (2020), arguing that ‘to effectively serve the goal of enhancing long-term shareholder value, corporate leaders should take into account stakeholder effects—as they should consider any other relevant factors’. In a similar way, adopting a group solution can enhance the long-term creditor value, and simultaneously benefit other stakeholders (e.g. employees, suppliers, distributors, society, the state (tax income)).

121 Kraakman et al. (2017), p 117.

122 World Bank, Principles for Effective Insolvency and Creditor/debtor Regimes (2015), C16.2. This recommendation has been preserved in the 2021 edition of the World Bank Principles for Effective Insolvency and Creditor/Debtor Regime, see C16.2.

123 UNCITRAL Legislative Guide, Part three (2010), Recommendations 211-213.

124 UNCITRAL Legislative Guide on Insolvency Law, Part four: Directors’ obligations in the period approaching insolvency (including in enterprise groups) (2019), Section two, para. 4, noting that the ‘short and long-term implications for the interests of the different enterprise group members may need to be assessed, which may involve accepting, even if only in the short term, some detriment to the interests of individual enterprise group members in order to achieve a longer term benefit for the enterprise group to which those individual members belong.’

125 EBA, Guidelines specifying the conditions for group financial support under Art. 23 of Directive 2014/59/EU, 9 July 2015, EBA/GL/2015/17.

126 Bork (2017), p 13.

127 Pottow (2006).

128 See Art. 38(1)(c) of the Statute of the International Court of Justice, listing among the sources of international law ‘the general principles of law recognized by civilized nations’.

129 Dworkin (1967), p 27.

130 Recent reforms of German insolvency law have led to the establishment of domestic rules on group coordination, similar to those of the EIR Recast. As explained by the commentators, the amended law did not pursue the goal of group consolidation, but instead was a result of endeavours to promote the spirit of coordination efforts. Fridgen et al. (2019).

131 Mevorach (2013), pointing out the variety of remedies available in group insolvencies and accepting that this may result in incoherency and uncertainty. Mevorach calls this ‘a realism of harmonization’.

132 UNCITRAL, Legislative Guide on Insolvency Law, Parts one and two (2004). World Bank, Principles for Effective Insolvency and Creditor/debtor Regimes (2015).

133 FSB, Key Attributes of Effective Resolution Regimes for Financial Institutions (2014).

134 Mokal (2007), p 58.
